# Prevalence of *Cytauxzoon felis* Infection-Carriers in Eastern Kansas Domestic Cats

**DOI:** 10.3390/pathogens9100854

**Published:** 2020-10-20

**Authors:** Yvonne M. Wikander, Tippawan Anantatat, Qing Kang, Kathryn E. Reif

**Affiliations:** 1 Department of Diagnostic Medicine/Pathobiology, College of Veterinary Medicine, Kansas State University, Manhattan, KS 66506, USA; ywikander@vet.k-state.edu (Y.M.W.); Tippawan@vet.k-state.edu (T.A.); 2 Department of Statistics, College of Arts and Sciences, Kansas State University, Manhattan, KS 66506, USA; qkang@k-state.edu

**Keywords:** *Cytauxzoon felis*, cytauxzoonosis, domestic cat, infection-carrier, PCR, prevalence, transmission reservoir

## Abstract

*Cytauxzoon felis* is a hemoprotozoal tick-transmitted pathogen of felids. Felids that survive acute disease often remain infected and serve as reservoirs for subsequent tick transmission to other susceptible felines. States adjacent to Kansas have identified *C. felis*-domestic cat carriers while statewide awareness and concern of cytauxzoonosis have increased. The objective of this study was to determine the prevalence of *C. felis-*carriers in the eastern Kansas domestic cat population using a sensitive quantitative PCR assay targeting the *C. felis* Cox3 mitochondrial gene. An overall *C. felis* infection prevalence of 25.8% was determined for asymptomatic domestic cats in eastern Kansas. Significantly more *C. felis*-carrier cats were identified in spring and fall, suggesting a seasonal fluctuation of survivors. Additionally, a greater percentage of feral and owned cats were positive for *C. felis* compared to rescue/rescinded cats. This study demonstrates that *C. felis*-domestic cat carriers are common among cats that spend at least a portion of time outdoors in eastern Kansas, and that more cats likely survive cytauxzoonosis than expected. Understanding the role of domestic cat carriers of *C. felis* is essential in developing cytauxzoonosis mitigation strategies, including recommending year-round use of acaricide products for all cats that spend any time outdoors.

## 1. Introduction

**Figure 1:**
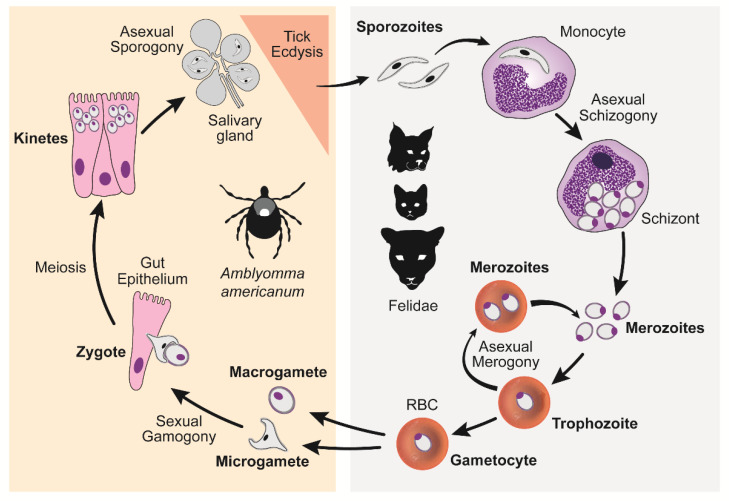
*C. felis* lifecycle. The right panel demonstrates asexual reproduction occurring within the host felid, while the left panel demonstrates the sexual and asexual reproduction occurring within the tick transmission vector.

*Cytauxzoon felis* is a tick-borne hemoprotozoal pathogen of felids and the agent of cytauxzoonosis, an often fatal disease of domestic cats in the southeastern and south-central United States (U.S.). *C. felis* has a complex lifecycle that includes an asexual stage within a felid host and a sexual reproductive stage within a competent ixodid tick vector [1] (Figure 1). Briefly, *C. felis* sporozoites are transmitted to a felid via tick saliva during a blood meal. Within the felid host, sporozoites enter monocytes and begin a schizogenous, or leukocyte, phase of asexual replication resulting in the release of many 1–2 µm diameter signet ring merozoites. Merozoites enter host erythrocytes and either replicate asexually via merogony or develop into gametocytes. Ticks become infected again by ingesting gametocytes when feeding on a felid carrier host. In the lumen of the tick gut, sexual reproduction occurs among gametocytes resulting in the formation of zygotes. Zygotes invade the gut epithelium, transform into kinetes, migrate to the salivary gland, and transform into sporozoites, the life stage dispensed via saliva during the subsequent tick bloodmeal. Successful transmission of infectious sporozoites requires transstadial maintenance of the parasite through larvae-to-nymph or nymph-to-adult ecdysis. In the U.S., competent biological transmission vectors for *C. felis* include the ixodid ticks *Dermacentor variabilis* (American dog tick) and *Amblyomma americanum* (Lone star tick) [2,3,4,5]. The geographic distribution of *D. variabilis* extends throughout the eastern half of the U.S. and in focal regions along the western coast [6,7]. The geographic distribution of *A. americanum* largely overlaps with *D. variabilis* in the eastern U.S.; however, *A. americanum* occurs more densely in southeastern and mid-central regions [8,9]. Juvenile *D. variabilis* life stages prefer to feed on small to medium-sized rodents, while adults feed on variety of medium to large mammals including cats. In contrast, *A. americanum* is more indiscriminate in host choice, and both juvenile and adult life stages will aggressively seek out and feed upon a wide diversity of small to large mammals including cats. Due to its aggressive and non-discriminating nature where cats may serve as a host for any life stage, *A. americanum* is likely a more significant vector for *C. felis* in Kansas than *D. variabilis*.

The vertebrate intermediate hosts of *C. felis* are members of the family Felidae. In the U.S., clinical cases of cytauxzoonosis typically peak in late spring, with a smaller peak in early fall, corresponding with adult and nymph *A. americanum* life stage activity [10]. Acute cytauxzoonosis clinical signs appear during *C. felis* schizogenous asexual replication, which can cause severe illness and death [2,11]. Domestic cats with this disease frequently present with lethargy, anorexia, depression, fever, dehydration, icterus, pale mucous membranes, and splenomegaly [2,12,13], which often culminates in death two–three weeks post-infection due to hypoxic and proinflammatory cytokine-mediated injury of multiple organs, interstitial pneumonia, and disseminated intravascular coagulation [14,15,16,17]. If presented early enough to initiate a ten-day treatment with atovaquone (15 mg/kg) and azithromycin (10 mg/kg), up to 60% of patients may recover [18]. Cats that survive the schizogenous phase of the parasite’s life cycle become asymptomatic, persistently parasitemic infection carriers, contributing to continued *C. felis* transmission to other felids via tick vectors [19]. *Cytauxzoon* spp. infections with similar clinical signs and disease progression have also been reported in Asia, South America, and Europe [20,21,22,23].

Several felid species have been documented as natural *C. felis* infection reservoirs, including: bobcat (*Lynx rufus*) [24,25,26,27,28,29], domestic cat (*Felis catus*) [30,31], cougar (*Puma concolor*) [32,33,34], and captive tigers [35]. For many decades, bobcats were presumed the main infection reservoir, while domestic cats were considered a dead-end host as those with observed clinical disease commonly died [11,36,37]. Recent *C. felis* transmission and prevalence studies however, challenge the domestic cat dead-end host assumption [19], with a combination of studies experimentally demonstrating and others documenting domestic cats as competent *C. felis*-carriers [2,38,39].

Several studies evaluating the distribution and prevalence of *C. felis* wild and domestic felid reservoirs have been conducted in the U.S. One study examining *C. felis* prevalence in wild felids found 20% (138/696) of bobcats over 14 states positive, with 79%, 65%, and 31% of sampled bobcats positive for *C. felis* in Missouri, Oklahoma, and Kansas, respectively [29]. Surveys of domestic cat populations demonstrate that *C. felis* prevalence can vary widely among these felid populations. Evaluation of *C. felis* infection among trap-neuter-release cats in Florida, North Carolina, and Tennessee identified a 0.3% (3/961) overall prevalence [40], similar to another study that observed a 0.8% (3/380) *C. felis* infection prevalence among healthy free-roaming cats in Oklahoma [41]. The latter study also evaluated cats in Iowa but did not identify *C. felis* infection in any of the 292 free-roaming cats tested in that state. However, another study identified 6.2% (56/902) of healthy client-owned cats infected with *C. felis* in Arkansas (15.5%, 25/161), Missouri (12.9%, 8/62), and Oklahoma (3.4%, 23/679) [39].

Intensifying and expanding *A. americanum* populations, heighted public awareness and concern over cytauxzoonosis, and anecdotal reports of increasing cytauxzoonosis cases in eastern Kansas suggest that domestic cat reservoirs may be contributing to local *C. felis* transmission cycles. The prevalence of *C. felis*-domestic cat carriers in Kansas is unknown; however, studies in adjacent states (Oklahoma and Missouri) have identified 0.8–12.9% of domestic cats may be *C. felis*-carriers [39]. The objective of this study was to determine the prevalence of *C. felis-*carriers in the domestic cat population of eastern Kansas by obtaining blood samples from asymptomatic, predominantly outdoor cats (feral, rescue/rescinded, and/or owned), and evaluating samples for *C. felis* infection using a quantitative PCR (qPCR) assay targeting the multi-copy cytochrome oxidase 3 (Cox3) gene [42]. Based on previous lower Kansas bobcat *C. felis*-carrier prevalence and adjacent state domestic cat *C. felis*-carrier prevalence studies, we hypothesized: (i) the prevalence of *C. felis* reservoirs in eastern Kansas domestic cats would be less than that found in Missouri and Oklahoma cats; (ii) chronic *C. felis* infection prevalence would be stable despite collection season; and (iii) a greater percentage of feral cats would be carriers compared to rescued or owned cats. Knowing the prevalence of *C. felis*-carriers among local domestic cat populations is important for developing and recommending cytauxzoonosis transmission mitigation strategies.

## 2. Results

### 2.1. Cox3 Mitochondrial DNA (miDNA) qPCR Assay Limit of Detection (LOD) and Limit of Quantification (LOQ)

Using blood from a cat acutely infected with *C. felis*, a series of assays was conducted to determine the LOD for the Cox3 qPCR assay used to evaluate domestic cat blood samples for *C. felis* infection in this study. The highest dilution of acute infected cat blood which consistently tested positive using the Cox3 qPCR assay was 1:10^5^, while still producing an amplicon-specific melt temperature of 76 °C ± 1 °C. Using the described Cox3 qPCR assay conditions, reagents, and equipment, a mean Ct value of 38.78 (95% CI 37.14–40.40) can reliably be interpreted as positive for *C. felis* assuming a correct melt peak is also detected (Table 1).

An LOQ study was performed for the Cox3 qPCR assay to determine the lowest copy number of the *cox3* target that could be reliably quantified from baseline with acceptable accuracy. The lowest dilution of amplicon-containing plasmid consistently detected using the Cox3 qPCR assay was 10^2^ with a melt temperature of 76 °C ± 1 °C. From the LOQ study, using the described Cox3 qPCR assay conditions, reagents, and equipment, the lowest number of the *cox3* target that can be reliably quantified from baseline with acceptable accuracy is 12.46 copies per μL (95% CI 9.12–15.80) (Table 2).

### 2.2. C. felis Infection Prevalence in Domestic Cats in Eastern Kansas

**Figure 2:**
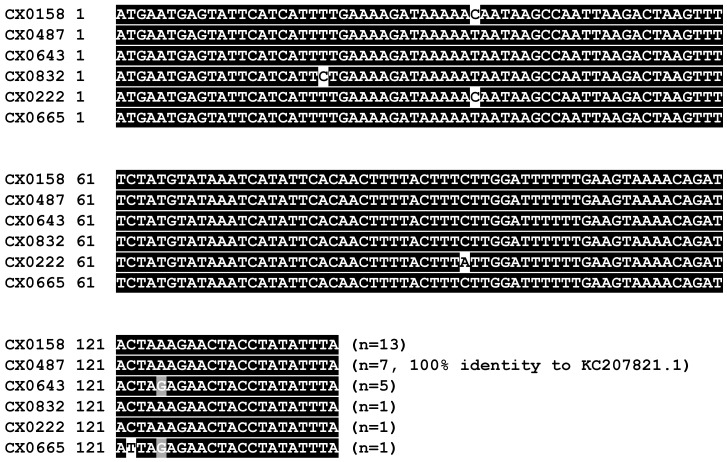
Alignment of representative unique *C. felis cox3* amplicon sequences. Alignment of six unique *C. felis cox3* 142 bp amplicon sequences. Frequency of sequence recovery from 28 sequenced samples provided after sequences. The second most common sequence was identical to *C. felis* GenBank Accession #: KC207821.1.

A total of 1131 blood samples from domestic cats were received from May 2018 through November 2019. Twenty-seven samples were excluded from the study because they were collected from cats acutely ill with cytauxzoonosis (n = 7) or from cats from neighboring states (n = 20), leaving 1104 samples from domestic cats with no known history of cytauxzoonosis from eastern Kansas available for *C. felis* infection carrier evaluation. In total, 270 of 1104 cats tested positive for *C. felis* (Table S1), resulting in a domestic cat *C. felis*-carrier prevalence of 25.8% (95% CI 23.7–27.9%) in eastern Kansas. To confirm the *C. felis* infection results, qPCR amplicons from approximately 10% (n = 28) of positive samples were cloned and sequenced. All sequenced amplicons shared 98.6 to 100% identity with *C. felis* GenBank Accession Number KC207821.1. Alignment of the 142 bp internal primer region produced six unique amplicon sequences (Figure 2).

**Figure 3:**
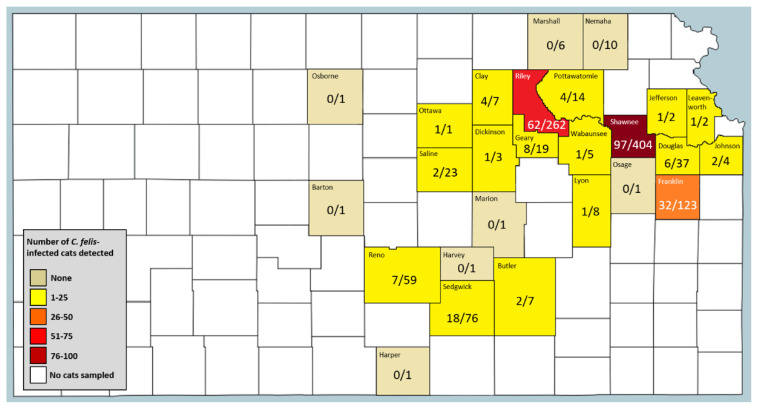
County-level location of identified *C. felis*-carrier domestic cats in Kansas. *C. felis*-carrier cats were identified in colored counties, with color legend indicating the overall incidence of *C. felis*-carriers identified from that county. No cats were evaluated from unshaded counties. Numbers represent raw *C. felis*-infected cat counts in the numerator with total cat blood samples obtained from that county in the denominator.

County-level locations of identified *C. felis*-carrier cats in Kansas are presented in Figure 3. The number of samples received from each county varied (Figure 3), with >100 samples received from three counties (Riley, Shawnee, Franklin) representing 11.5% of total samples, and <10 samples received from 16 counties. Of the 26 eastern Kansas counties from which cat blood samples were received, *C. felis*-infected blood samples were detected from cats in 18 counties. Statistical analysis of location data for reservoir population in specific counties was not possible because the sampling method across counties was not standardized as samples were opportunistically collected from cats undergoing routine procedures or bloodwork unrelated to our study.

### 2.3. Seasonal Variation of Detecting C. felis-Carrier Cats

**Figure 4:**
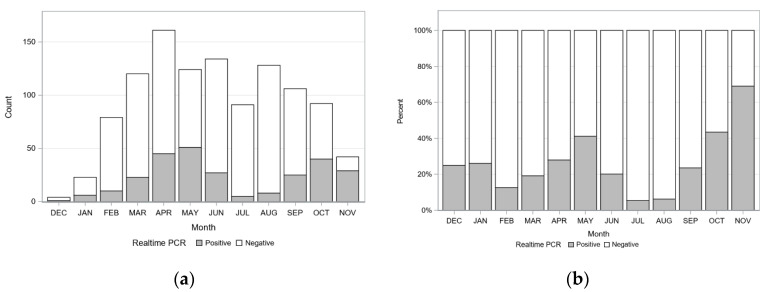
Number and percent of *C. felis*-carrier cats identified by collection month. (**a**) Raw counts of the number of *C. felis*-infected cats (gray bars) among total cats tested (white bars). (**b**) Percentage of cats *C. felis*-infected (gray bars) among total tested by month (white bars).

**Figure 5:**
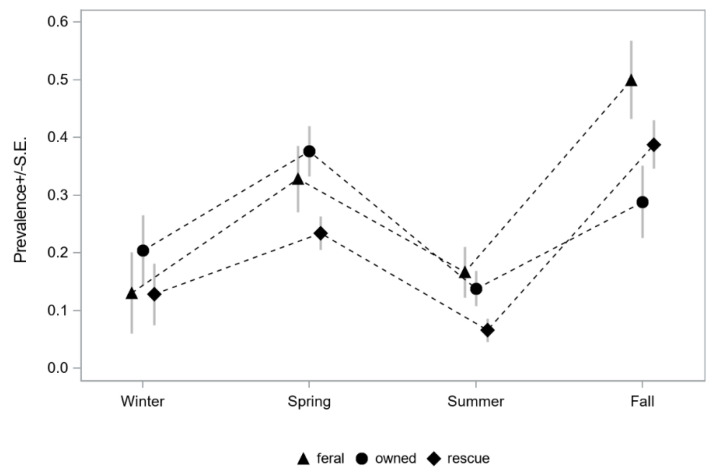
*C. felis* among different cat lifestyle populations and season. Lifestyle is indicated by the triangle for feral cats (triangle), owned cats (circle), and rescue/rescinded (diamond) cats.

Incidence of detecting *C. felis*-carrier cats during different months or seasons was investigated. The number of cats tested and the number of cats testing positive for *C. felis* are presented in Figure 4 (Table S2, Figure S1). The greatest number of *C. felis*-carrier cats was observed in April (n = 45), May (n = 51), and October (n = 40), with the greatest percentage of *C. felis*-carrier cats observed in May (41.1%), October (43.5%), and November (69.0%). Since the number of cats tested varied from month to month, testing results were evaluated by season: winter, spring, summer, and fall. A significant seasonal pattern for identifying *C. felis*-carrier cats was observed (*p* < 0.001). A significantly greater percentage of *C. felis*-carrier cats were detected in fall compared to all other seasons, and in spring compared to summer and winter (Table 3, Figure 5).

### 2.4. Evaluation of C. felis-Carriers among Cats with Different Lifestyles

The number (percentage) of individual blood samples obtained from cats with different lifestyles was (i) 537 (48.6%) rescue/rescinded cats from shelter/humane society/rescue organizations, (ii) 351 (31.8%) privately owned cats presenting to veterinary clinics, and (iii) 216 (19.6%) feral cats from catch-and-release programs (Table S3). Overall, *C. felis* infection prevalence significantly varied by cat lifestyle (*p* = 0.007). Of these different lifestyles, 21.8% (117/537) of rescue cats, 25.4% (89/351) of owned cats, and 29.6% (64/216) of feral cats were infected with *C. felis* (Table 4). Analyzing *C. felis* infection data by cat lifestyle, feral and owned cats were 1.7 and 1.5 times, respectively, more likely to be *C. felis*-carriers than rescue cats (Table 4). The prevalence of *C. felis*-carriers among feral and owned cats was not significantly different (Table 4). A similar seasonal trend in detecting *C. felis*-carriers among cats from lifestyles was observed, with more *C. felis*-carriers observed from cats sampled in the spring and fall (Figure 5, Table S2).

## 3. Discussion

Acute cytauxzoonosis is one of the most fatal diseases in domestic cats, such that for a long time domestic cats were thought to have a minimal, if any, role in the enzootic maintenance of *C. felis* [11,18,32]. Over the past two decades, several prevalence studies have confirmed domestic cats as *C. felis* reservoirs [39,40,41] and experimentally demonstrated that domestic cats can serve as competent reservoirs for subsequent *C. felis* transmission by ticks, especially *A. americanum* [3,4,38]. *C. felis*-carrier surveys in states adjacent to Kansas have identified that 0.8–12.9% of domestic cats may be *C. felis*-carriers [39,41]. Anecdotal reports of increasing cytauxzoonosis cases and an abundant population of *A. americanum* in eastern Kansas suggest that domestic cat carriers may be contributing to the increased attention for this disease. In the present study, we demonstrate: (1) there is a high prevalence of asymptomatic *C. felis-*domestic cat carriers in eastern Kansas, (2) *C. felis*-domestic cat carriers are more common during spring and fall months, and (3) feral and owned cats are more likely to be *C. felis*-carriers than rescue/rescinded cats.

The prevalence of active *C. felis* parasitemia identified in this study was significantly greater than that determined in previous surveys conducted in neighboring states. However, direct comparisons of our findings with those of other studies is difficult as different assay platforms (conventional versus quantitative PCR), gene targets (ITS-1 rRNA, 18S rRNA, Cox3 miDNA), and gene target copy numbers (single-copy, multi-copy) were used to detect *C. felis* infection [29,39,40,41]. Although most *C. felis* prevalence studies involving domestic cats have used conventional or nested endpoint PCR assays targeting the 18S rRNA *C. felis* gene [13,40,41,43], a recent study demonstrated that a Cox3 miDNA real-time PCR assay performed with similar sensitivity to the commonly used 18S rRNA endpoint PCR assay in detecting *C. felis* in chronically infected cats [42]. For this study, we chose to use the published *C. felis* Cox3 qPCR assay [42] because it targeted a highly conserved multi-copy gene, and greatly improved testing efficiency while retaining a level of sensitivity similar to previous endpoint PCR assays. A sensitive and specific assay was important as parasitemia levels are often low in reservoir hosts. The specific *cox3* copy number per *C. felis* parasite is unknown and could possibly vary in different *C. felis* developmental stages. Additionally, whether *C. felis* parasitemia levels remain stable, cyclically vary like other tick-borne pathogens, or slowly wane during persistent infection of reservoir hosts is unknown. Differences in molecular assay target copy number during different pathogen developmental stages or fluctuating parasitemia in carrier cats could affect identification of reservoir hosts.

Of note, our study and previous *C. felis* infection surveys used molecular assays to assess infection prevalence, compared to many other tick-borne pathogen surveys of companion animals which commonly use serologic-based diagnostic assays. This difference is notable because a positive molecular test result provides evidence of likely active infection (pathogen must be present in order to detect its genetic material) compared to serologic-based assays which evaluate the presence of a pathogen-specific host antibody (host may or may be not be actively infected). Currently, no serologic assays are commercially available to detect *C. felis* infection or exposure in domestic cats.

Once thought to play a minimal or no role in the enzootic maintenance of *C. felis*, domestic cats are now being recognized as likely important *C. felis* infection reservoirs [39,40,44]. Our findings support that domestic cats are not only a competent reservoir of *C. felis* but may play a prominent role in local enzootic maintenance, with 25% of surveyed cats positive for *C. felis* infection. This large percentage of *C. felis*-carrier cats suggests that the mortality rate for this disease is less than previously thought, or that multiple *C. felis* strains of varying virulence are circulating in eastern Kansas. *C. felis* strain may also impact treatment success as one study evaluating the efficacy of atovaquone and azithromycin treatment observed greater treatment success and improved survival in cats infected with *C. felis* with a specific cytochrome b genotype [43]; however, other genetic loci were not predictive of treatment success [18]. While genetic variation among *C. felis* is described [45,46,47,48], how virulence or treatment success varies for different *C. felis* strains/isolates is largely unexplored. Additional studies evaluating genetic differences among cats that survive or succumb to *C. felis* infection will be needed. However, a significant roadblock to conducting studies that could address these questions is the lack of in vitro assay systems and isolate/strain repositories.

The significant seasonal variation pattern of detecting *C. felis*-carriers was surprising as we anticipated that as long-term or chronic infection carriers, there would be minimal to no seasonal influence (e.g., once infected, the cat stays infected). The observed seasonal fluctuation in carrier cats could represent a new influx of cats recently recovered from the schizogenous phase that correlates with seasonal tick vector activity or possibly a seasonal fluctuation in carrier cat parasitemia. Whether parasitemia remains consistent, cycles, or slowly wanes overtime in response to ongoing merogony cycles, seasonality, and/or host immune responses is unknown. Cats that have survived a schizogenous *C. felis* phase are thought to be immune to clinical disease associated with additional *C. felis* challenge [1,49,50]. Although these cats may not develop clinical signs of disease, whether re-exposure/infection, especially with a different *C. felis* strain, impacts parasitemia is also unknown. A study evaluating the prevalence of *C. felis* in southern Illinois wild-caught bobcats identified one individual that appeared to have been infected with a different strain (ITS-1 single nucleotide polymorphism) of *C. felis* between captures [28]. More recently, a domestic shorthair cat successfully treated for acute cytauxzoonosis seven years prior presented with a repeat acute *C. felis* infection confirmed on splenic histology [51]. Evidence of *C. felis* seasonal variation among cat carriers, susceptibility to co- or sequential infections of different *C. felis* strains, and the possibility of recrudescent or newly acquired infection leading to additional clinical disease suggests that *C. felis*-carrier cats may both contribute to sustained pathogen transmission while still remaining vulnerable to additional disease events.

As expected, feral cats had the greatest prevalence of *C. felis* infection. Feral cats are 100% outdoors, likely encounter large numbers of ticks, and are least likely to be treated with any sort of acaricide product, all increasing the *C. felis* infection risk and reservoir potential. Interestingly, although slightly lower, owned cats were statistically as likely to be *C. felis*-carriers, likely highlighting how pervasive *A. americanum* populations are in eastern Kansas. Rescue cats were significantly the least likely to be *C. felis*-carriers. This may be due to rescue cats typically being younger, and a tendency for rescue facilities to keep them indoors. One of our study limitations was that we were unable to collect additional background details on our study subjects including age, sex, detailed lifestyle data (e.g., percent of time spent outdoors), type of outdoor environment (e.g., woods, pasture, manicured yard), and acaricide usage. The only population of subjects that we could be sure spent significant time outdoors was the feral catch-and-release cats. An unknown percentage of pet cats presented to the veterinary clinics and rescue/rescinded cats may have had an unreported indoor-exclusive lifestyle. If so, then our prevalence rate would be artificially decreased for those populations. Additional studies will be needed to more comprehensively evaluate specific risk factors associated with *C. felis* infection and reservoir risk.

Most of the blood samples in this study came from three counties, with far fewer from the remaining 23 counties from which samples were obtained. As such, analysis of whether *C. felis* reservoir cat populations were more or less common in specific counties could not be performed. Only one previous study identified location-based differences in *C. felis*-carrier cat prevalence. Rizzi et al. determined that cats from eastern Oklahoma had a higher prevalence of *C. felis*-carriers than those from central Oklahoma and suggested this difference could be due to strain or virulence differences [39]. Meanwhile, Raghavan et al. concluded that the main risk factor for *Cytauxzoon* infection in cats, regardless of location, was land cover and humidity that favor the tick vector [52]. The prevalence of *C. felis*-carrier cats in specific areas is likely highly nuanced and dependent on several of the abovementioned variables. Further studies gathering samples using a standardized sampling method across counties are needed to more accurately assess if domestic cat *C. felis* reservoirs are more common in specific Kansas counties.

## 4. Conclusions

We determined the prevalence of *C. felis*-carrier domestic cats in eastern Kansas to be 25.8% (95% CI 23.7–27.9%). These findings suggest *C. felis* infection in domestic cats is far more prevalent than previously thought with many cats surviving the acute phase of the disease. These persistently parasitemic individuals then act as a disease reservoir for other felids. Studying *C. felis* is challenging in many ways as complex factors affect each stage of the organism, the tick vector, the host felid, and the environment, not to mention the lack of an in vitro study system. As such, all cats living in *C. felis* endemic areas, including indoor-exclusive cats, should be aggressively treated with acaricides known to prevent or minimize tick transmission per manufacturer recommendations. More studies are needed to better understand factors affecting *C. felis* and other *Cytauxzoon* spp. infections within the U.S. and globally and their outcomes.

## 5. Materials and Methods

### 5.1. Study Design

A prospective study with individual cats as the experimental units was designed. Inclusion criteria included: (i) any cat old enough to have been outdoors during one tick season (March–November); (ii) healthy and not exhibiting overt signs of clinical disease; and (iii) from eastern Kansas. Exclusion criteria included: (i) any cat displaying overt signs of clinical disease; or (ii) not from eastern Kansas. Data collected from each cat blood sample included: collection date (year/month/day); collection organization; collection location (city/county); and felid lifestyle (feral, rescue/rescinded, owned).

### 5.2. Blood Sample Sources and Collection

All study activities involving animals were performed in accordance with an approved Kansas State University Institutional Animal Care and Use protocol, approved prior to study initiation. To collect blood samples, participation was solicited from 28 humane societies, animal shelters/rescues, and small animal practices in eastern Kansas. Eight organizations agreed to participate and six submitted samples from May 2018 through November 2019 (Table S4). EDTA blood samples (up to 2.0 mL) were opportunistically collected from domestic cats presented for routine procedures or illness unrelated to cytauxzoonosis at veterinary clinics, animal shelters/rescues/humane societies, or feral cat catch-and-release programs. Some samples were used for other blood tests prior to submission for this study. All samples submitted were stored at −20 °C prior to shipment to Kansas State University for study testing.

EDTA whole blood samples were defrosted at room temperature for 10–15 min within three days of arrival and vortexed for 15 s to mix. A 100 µL aliquot of blood was reserved for genomic DNA extraction and up to 1 mL of blood was saved for additional future testing.

### 5.3. DNA Extraction

Total DNA was extracted from 100 µL of whole blood using the Quick-DNA^TM^ Miniprep Kit according to manufacturer’s instructions (Zymo Research, Irvine, CA, USA), with a final 35 µL elution volume. Extracted gDNA samples were stored at −20 °C.

### 5.4. Quantitative Real-Time PCR (qPCR)

Since *C. felis*-carrier cats can have very low parasite loads [19] and over 1000 samples were anticipated, a qPCR-based assay was optimal for efficient sample processing. Thus, a sensitive qPCR assay targeting a 192 bp region *C. felis* cytochrome c oxidase subunit III (Cox3) gene [42] was chosen to evaluate samples for *C. felis* infection. PCR mastermix setups were prepared in a PCR template-clean workstation and gDNA template was added to individual reaction mixtures in a different room. Each PCR reaction mixture consisted of 12.5 µL of 2XSsoAdvanced Universal SYBR Green Supermix (Bio-Rad Laboratories, Hercules, CA, USA), 1.25 µL of 10 µM *Cf* Cox3F (5′-GCATATCTTCAAATTACAGATACAC-3′), 1.25 µL 10 µM *Cf* Cox3R (5′-CCAGTAACTGTTTAGTGTAGTTAAC3′), 5 µL nuclease-free water, and 5 µL template for a 25 µL total reaction volume. Template consisted of DNA from blood samples, nuclease-free water as no template control (NTC), 1:100 gDNA from a clinical cytauxzoonosis cat blood sample (positive control), or 10-fold serial dilutions of pCR4-TOPO vector containing 192 bp *C. felis cox3* amplicon (standard curve). Samples were run on a CFX Connect Real-Time System (Bio-Rad Laboratories) using the following cycling parameters: initial denaturation at 98 °C for 3.5 min; 45 cycles of 60 °C for 30 s, 98 °C for 20 s; and a final melt curve (65–95 °C in 0.5 °C increments). The CFX Maestro Software (Bio-Rad Laboratories) was used to display results.

### 5.5. C. felis Cox3 qPCR Assay Limit of Detection (LOD)

Genomic DNA was extracted from the blood of a cat that had died from acute cytauxzoonosis and was diluted to a 1:100 concentration in DNA elution buffer. The Cox3 qPCR assay was performed as described above. Eight 10-fold serial dilutions of the 1:100 diluted acute cat blood were prepared using uninfected cat blood (obtained from Kansas State University’s Veterinary Teaching Hospital blood donor cat colony) as diluent. Genomic DNA was extracted as previously described. A similar procedure was performed using DNA elution buffer as diluent instead of uninfected cat blood. Duplicate PCR plates were assembled which included, in triplicate, 5 µL of the eight dilutions of DNA-extracted uninfected cat blood, 5 µL of the eight dilutions of elution buffer, 5 µL of nuclease-free water as NTC, and 5 µL of the 1:100 extracted infected cat blood as positive control. Cycling conditions were performed as previously described.

### 5.6. C. felis Cox3 qPCR Assay Limit of Quantification (LOQ)

*C. felis* Cox3 amplicon-containing plasmid with a known concentration of 10^5^ copies per μL DNA was used. The same process described for the above LOD was followed except for replacing the extracted infected cat DNA with the Cox3 plasmid and producing duplicate 10-fold serial dilutions a total of five times, resulting in a final dilution of 10^0^ copies per μL DNA. A PCR plate was assembled which included duplicate sets of triplicate samples consisting of 5 µL of the five plasmid dilutions in DNA-extracted uninfected cat blood, 5 µL of the five plasmid dilutions of elution buffer, 5 µL of 10^5^ copies per μL DNA *C. felis* Cox3 plasmid as positive control, three samples of 5 µL of nuclease-free water as NTC, and three samples of uninfected DNA-extracted cat blood. Cycling conditions were performed as previously described.

### 5.7. Amplicon Cloning and Sequence Analysis

To confirm amplicon identity, the resulting amplicon from approximately 10% of *C. felis cox3*-positive qPCR reactions was cloned in a pCR4-TOPO ™ cloning vector and transformed into TOP10 *E. coli* according to the manufacturer’s instructions (Invitrogen, Carlsbad, CA, USA). A minimum of five resultant clones were screened for presence of a correctly sized insert and a minimum of two positive clones were submitted for rolling cycle amplification (RCA) and 5′ and 3′ sequencing using T3 or T7 sequencing primers (MC Lab, South San Francisco, CA, USA). The 142 bp region internal to the *C. felis cox3* primers was aligned using MEGA-X and alignments were presented using BoxShade v3.2.

### 5.8. Statistical Analyses

PCR results for samples were treated as binary responses indicating the presence or absence of *C. felis* infection. These binary data were analyzed under the logit linear model. The full model includes season (winter (December to February), spring (March to May), summer (June to August), fall (September to November)), lifestyle (feral, owned, rescue), and their interaction as the fixed effects (Table S5, Table S6). Since the interaction was not significant (*p*-value = 0.13), the full model was simplified to a reduced model containing main effects of season and lifestyle only to quantify prevalence rates. The goodness-of-fit of the reduced model was verified based on the deviance-to-degrees-of-freedom ratio of 1.73 with a *p*-value of 0.109. Prevalence rates for each season were calculated by weighting model estimates according to the Kansas cat population of 49.9% for feral, 47.1% for owned, and 3.0% for rescue cats [53,54,55,56,57]. Tukey’s multiplicity adjustment was applied when comparing among the four levels of season effect and among the three levels of lifestyle. Prevalence rates for each lifestyle were calculated by weighting model estimates according to 1:1:1:1 allocation of winter, spring, summer, and fall season. Moreover, the overall prevalence rate was calculated based on 49.9%:47.1%:3.0% allocation of feral, owned, and rescue cats as well as 1:1:1:1 allocation of the four seasons. Statistical analysis was performed using the LOGISTIC procedure of the SAS/STAT^®^ software, Version 9.4 (SAS Institute Inc., Cary, NC, USA).

## Figures and Tables

**Table 1:** *C. felis* Cox3 qPCR assay limit of detection (LOD).

Sample Conc	Ct ^1^ Value	Melt Temp ^2^ (°C)
1:10^5^	40.36	75.50
1:10^5^	37.62	75.00
1:10^5^	41.11	75.50
1:10^5^	40.69	75.50
1:10^5^	37.10	75.00
1:10^5^	35.77	75.00

^1^ Number of amplification cycles required to reach a fixed cycle threshold where the fluorescent signal exceeds the background level. ^2^ Temperature at which double-stranded DNA template disassociates into single strands.

**Table 2:** *C. felis* Cox3qPCR assay limit of quantification (LOQ).

Sample Conc	Ct ^1^ Value	SQ ^2^	Melt Temp ^3^ (°C)
10^2^	38.81	6	75.50
10^2^	37.00	18	75.50
10^2^	38.21	9	75.50
10^2^	37.58	13	75.50
10^2^	37.53	13	75.50
10^2^	37.24	16	75.50

^1^ Number of amplification cycles required to reach a fixed cycle threshold where the fluorescent signal exceeds the background level. ^2^ Starting quantity (SQ) is the number of DNA copies at the beginning of the reaction. ^3^ Temperature at which double-stranded DNA template disassociates into single strands.

**Table 3:** *C. felis* infection prevalence by season.

			Odds Ratio to (Adj. *p*-Value of Testing for Odds Ratio^ = 1)		
Season	Prevalence	SE ^1^	Spring	Summer	Fall
Winter	18.2%	4.0%	0.42 (0.016)	1.46 (0.624)	0.27 (<0.001)
Spring	34.4%	3.0%		3.44 (<0.001)	0.64 (0.048)
Summer	13.3%	2.0%			0.19 (<0.001)
Fall	45.1%	3.8%			
Overall	25.8%	2.1%			

^1^ Standard error.

**Table 4:** *C. felis* infection prevalence by cat lifestyle.

			Odds Ratio to (Adj. *p*-Value of Testing for Odds Ratio^ = 1)	
Lifestyle	Prevalence	SE ^1^	Owned	Rescue
Feral	27.2%	3.2%	1.12 (0.840)	1.70 (0.015)
Owned	25.0%	2.5%		1.51 (0.038)
Rescue	18.0%	1.8%		
Overall	25.8%	2.1%		

^1^ Standard error.

## Supplementary Materials

The following are available online at https://www.mdpi.com/2076-0817/9/10/854/s1, Table S1: *Cytauxzoon felis* prevalence by month and lifestyle, Table S2: *Cytauxzoon felis* prevalence by season and lifestyle, Table S3: Cat blood samples collected per season and lifestyle, Figure S1: Number and percent of *C. felis* reservoir cats identified by collection month and lifestyle, Table S4: Cat blood samples submitted by blood collector organization and lifestyle, Table S5: *C. felis* infection prevalence by lifestyle in each season, Table S6: *C. felis* infection season and lifestyle interaction test.
