# Supplementary material for: Prevalence of Cytauxzoon felis Infection-Carriers in Eastern Kansas Domestic Cats
Source: Pathogens. 2020 Oct 20;9(10):854. doi: 10.3390/pathogens9100854 (PMC7594093; doi:10.3390/pathogens9100854)
Supplement: Supplementary file 1 [file pathogens-09-00854-s001.zip › pathogens-942077-supplementary.docx]

| **Month** | **feral** | | | **owned** | | | **rescue** | | | **Total** | | |
| --- | --- | --- | --- | --- | --- | --- | --- | --- | --- | --- | --- | --- |
|  | **Prevalence** | **Incidence** | **# of Cats** | **Prevalence** | **Incidence** | **# of Cats** | **Prevalence** | **Incidence** | **# of Cats** | **Prevalence** | **Incidence** | **# of Cats** |
| **FEB** | 13.0% | 3 | 23 | 12.8% | 5 | 39 | 11.8% | 2 | 17 | 12.7% | 10 | 79 |
| **MAR** | 20.0% | 2 | 10 | 40.6% | 13 | 32 | 10.3% | 8 | 78 | 19.2% | 23 | 120 |
| **APR** | 31.0% | 13 | 42 | 23.9% | 11 | 46 | 28.8% | 21 | 73 | 28.0% | 45 | 161 |
| **MAY** | 46.7% | 7 | 15 | 48.9% | 23 | 47 | 33.9% | 21 | 62 | 41.1% | 51 | 124 |
| **JUN** | 34.4% | 11 | 32 | 15.5% | 9 | 58 | 15.9% | 7 | 44 | 20.1% | 27 | 134 |
| **JUL** | 9.1% | 1 | 11 | 7.3% | 3 | 41 | 2.6% | 1 | 39 | 5.5% | 5 | 91 |
| **AUG** | 0.0% | 0 | 29 | 19.4% | 6 | 31 | 2.9% | 2 | 68 | 6.3% | 8 | 128 |
| **SEP** | 13.0% | 3 | 23 | 28.8% | 15 | 52 | 22.6% | 7 | 31 | 23.6% | 25 | 106 |
| **OCT** | 75.0% | 9 | 12 | . | . | . | 38.8% | 31 | 80 | 43.5% | 40 | 92 |
| **NOV** | 78.9% | 15 | 19 | . | . | . | 60.9% | 14 | 23 | 69.0% | 29 | 42 |
| **JAN** | . | . | . | 80.0% | 4 | 5 | 11.1% | 2 | 18 | 26.1% | 6 | 23 |
| **DEC** | . | . | . | . | . | . | 25.0% | 1 | 4 | 25.0% | 1 | 4 |
| **Total** | 29.6% | 64 | 216 | 25.4% | 89 | 351 | 21.8% | 117 | 537 | 24.5% | 270 | 1104 |

**Table 1.** *C. felis* prevalence by month and lifestyle.

| **Season.** | **feral** | | | **owned** | | | **rescue** | | | **Total** | | |
| --- | --- | --- | --- | --- | --- | --- | --- | --- | --- | --- | --- | --- |
|  | **Prevalence** | **Incidence** | **# of Cats** | **Prevalence** | **Incidence** | **# of Cats** | **Prevalence** | **Incidence** | **# of Cats** | **Prevalence** | **Incidence** | **# of Cats** |
| **Winter** | 13.0% | 3 | 23 | 20.5% | 9 | 44 | 12.8% | 5 | 39 | 16.0% | 17 | 106 |
| **Spring** | 32.8% | 22 | 67 | 37.6% | 47 | 125 | 23.5% | 50 | 213 | 29.4% | 119 | 405 |
| **Summer** | 16.7% | 12 | 72 | 13.8% | 18 | 130 | 6.6% | 10 | 151 | 11.3% | 40 | 353 |
| **Fall** | 50.0% | 27 | 54 | 28.8% | 15 | 52 | 38.8% | 52 | 134 | 39.2% | 94 | 240 |
| **Total** | 29.6% | 64 | 216 | 25.4% | 89 | 351 | 21.8% | 117 | 537 | 24.5% | 270 | 1104 |

**Table 2.** *C. felis* prevalence by season and lifestyle.

**Table 3.** Cat blood samples collected per season and lifestyle.

| **Season** | **feral** | | **owned** | | **rescue** | | **Total**  **N^1^** |
| --- | --- | --- | --- | --- | --- | --- | --- |
|  | **N^1^** | **Row Percent** | **N^1^** | **Row Percent** | **N^1^** | **Row Percent** |  |
| **Winter** | 23 | 21.7% | 44 | 41.5% | 39 | 36.8% | 106 |
| **Spring** | 67 | 16.5% | 125 | 30.9% | 213 | 52.6% | 405 |
| **Summer** | 72 | 20.4% | 130 | 36.8% | 151 | 42.8% | 353 |
| Fall | 54 | 22.5% | 52 | 21.7% | 134 | 55.8% | 240 |
| Total | 216 | 19.6% | 351 | 31.8% | 537 | 48.6% | 1104 |

^1^ Number of cat blood samples.


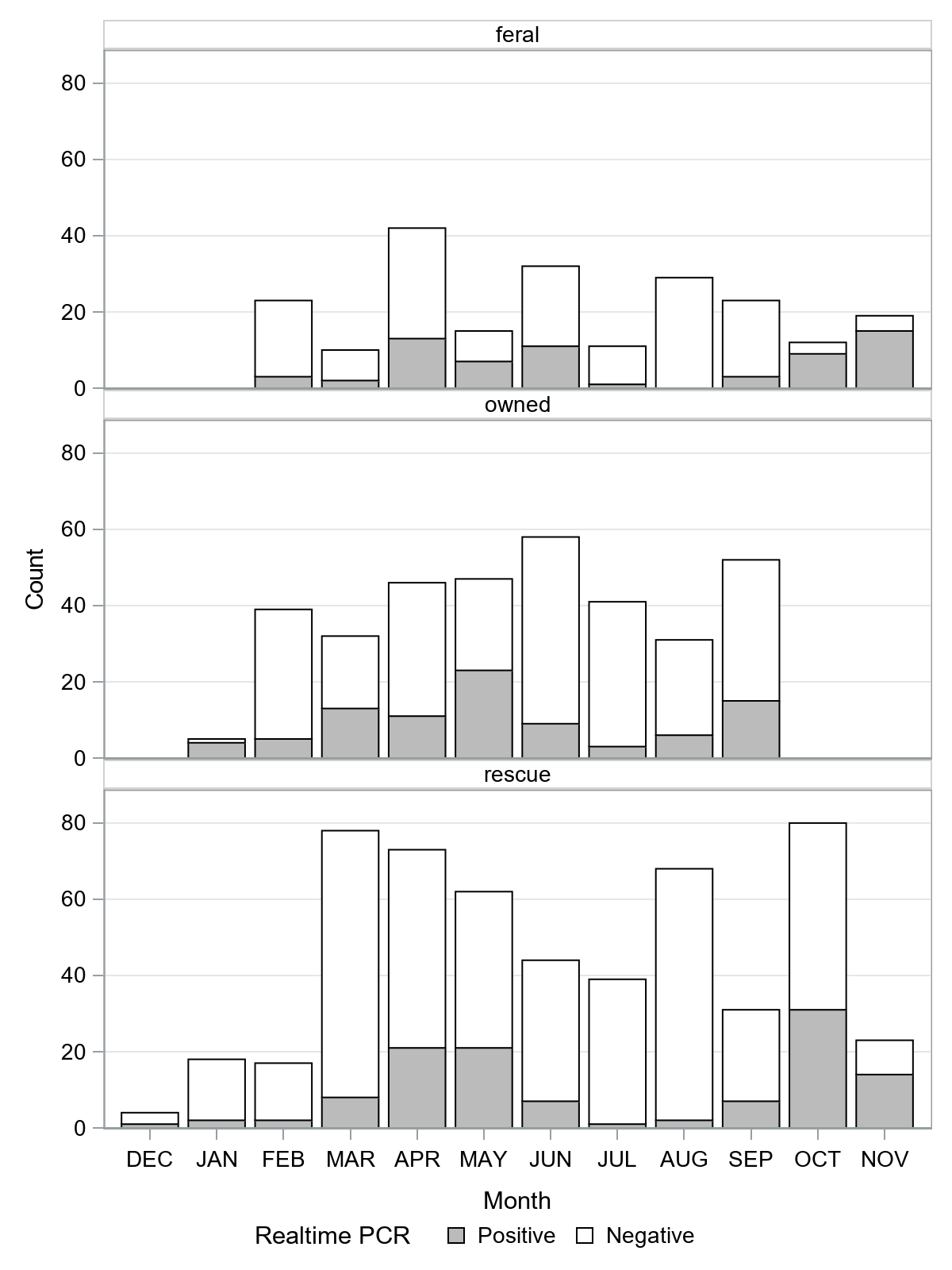

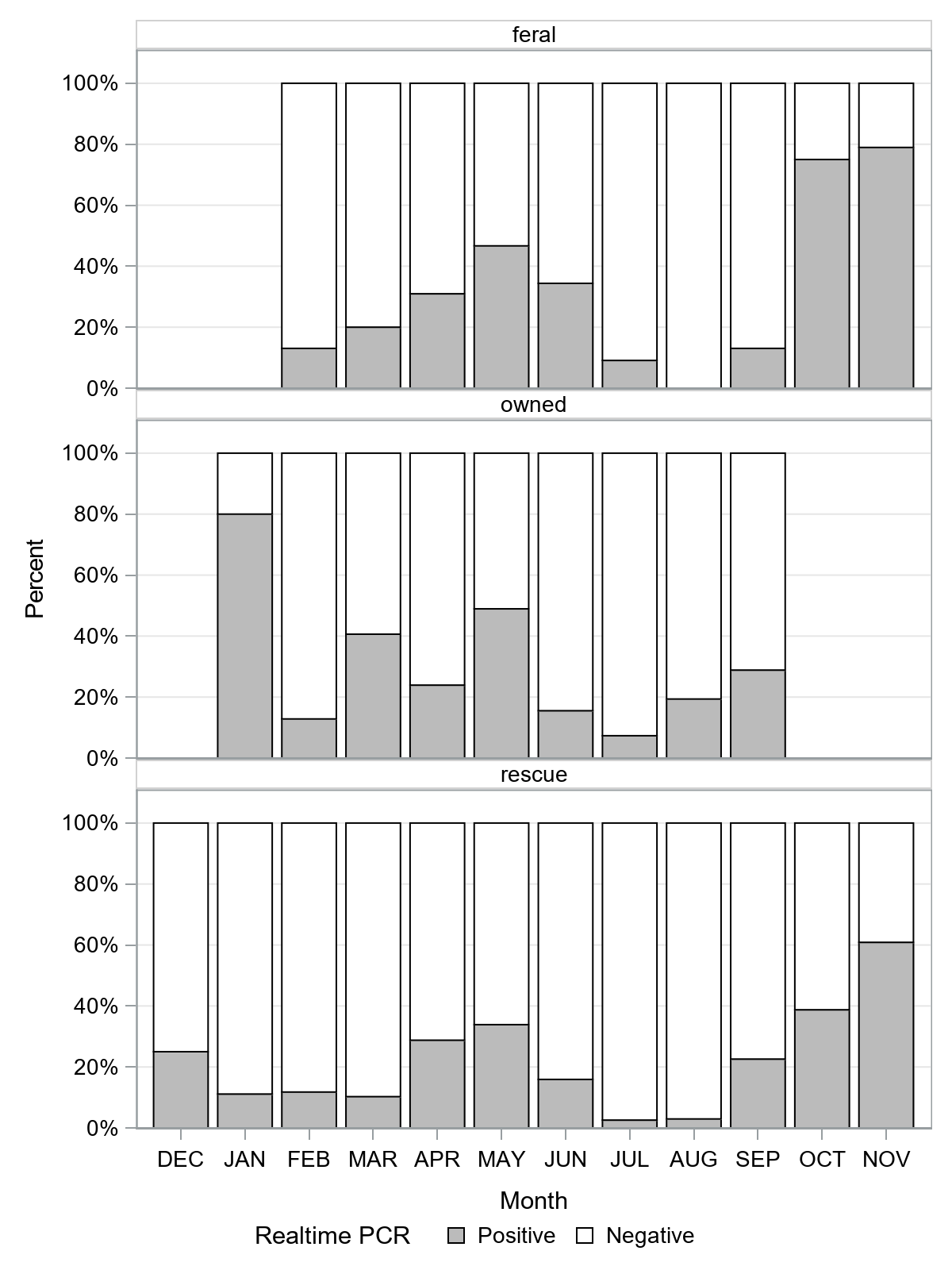


**Figure S1.** Number and percent of *C. felis* reservoir cats identified by collection month and lifestyle. (a) Raw counts of the number of *C. felis*-infected cats (gray bars) among total cats tested (white bars). (b) Percentage of cats infected with *C. felis* (gray bars) among total tested by month (white bars).

**Table 4.** Cat blood samples submitted by blood collector organization and lifestyle.

| **Sample**  **Submitter** | **Predominant**  **Location** | **County**  **Location** | **Samples**  **Submitted** | **Lifestyle** |
| --- | --- | --- | --- | --- |
| AD^1^ | Junction City | Geary | 5 | pet |
| BSVC^2^ | Sabetha | Bern | 9 | pet |
| KSVDL^3^ | Manhattan | Riley | 337 | pet |
| LHS^4^ | Lawrence | Douglas | 33 | rescue |
| HHHS^5^ | Topeka | Shawnee | 195 | rescue |
| KSUSM^7^ | Ottawa | Franklin | 309 | rescue |
|  | Manhattan | Riley |  |  |
|  | Hutchinson | Reno |  |  |
| KSUSM^7^ | Topeka | Shawnee | 216 | feral |
|  | Wichita | Sedgwick |  |  |

**^1^ Animal Doctor (Junction City, KS), ^2^ Bern-Sabetha Veterinary Clinic (Sabetha, KS), ^3^ Kansas State Diagnostic Clinical Pathology Laboratory (Manhattan, KS), ^4^ Lawrence Humane Society (Lawrence, KS), ^5^ Helping Hands Humane Society (Topeka, KS), and ^7^ KSU Shelter Medicine (**Manhattan, KS). Table 5. *C. felis*-infection prevalence by lifestyle in each season.

| **Season** | **Status** | **Prevalence** | **SE^1^** |
| --- | --- | --- | --- |
| Winter | feral | 13.0% | 7.0% |
|  | owned | 20.5% | 6.1% |
|  | rescue | 12.8% | 5.4% |
| Spring | feral | 32.8% | 5.7% |
|  | owned | 37.6% | 4.3% |
|  | rescue | 23.5% | 2.9% |
| Summer | feral | 16.7% | 4.4% |
|  | owned | 13.8% | 3.0% |
|  | rescue | 6.6% | 2.0% |
| Fall | feral | 50.0% | 6.8% |
|  | owned | 28.8% | 6.3% |
|  | rescue | 38.8% | 4.2% |

^1^ Standard Error.

**Table 6.** *C. felis*-infection statistical tests of season, lifestyle, and season-by-lifestyle interaction.

| **Effect** | **P-value** | |
| --- | --- | --- |
|  | **Full Model** | **Reduced (Final) Model** |
| season | <0.001 | <0.001 |
| lifestyle | 0.079 | 0.007 |
| season*lifestyle | 0.130 | -- |
